# 3-[1-(3,4-Dichloro­phen­yl)eth­yl]-1,3-thia­zinane-2-thione

**DOI:** 10.1107/S1600536809046352

**Published:** 2009-11-11

**Authors:** Fu-Feng Yan, Chong-Jia Liang

**Affiliations:** a Provincial Key Laboratory of Surface & Interface Science, Zhengzhou University of Light Industry, Zhengzhou 450002, People’s Republic of China; b Henan Sports School, Zhengzhou 450044, People’s Republic of China

## Abstract

In the title compound, C_12_H_13_Cl_2_NS_2_, the thia­zinane ring adopts a half-boat conformation. An intra­molecular C—H⋯S hydrogen bond is observed. In the crystal structure, centrosymmetrically related mol­ecules inter­act through an aromatic π–π stacking inter­actions, with a centroid–centroid separation of 3.790 (2) Å.

## Related literature

For the crystal structures of related thia­zinane compounds, see: Kálmán, *et al.* (1977 ▶); Peng & Wu (2009 ▶). For the biological activity of thia­zinane-containing compounds, see: Soloway *et al.* (1978 ▶); Tomizawa *et al.* (1995 ▶). For ring puckering parameters, see: Cremer & Pople (1975 ▶).
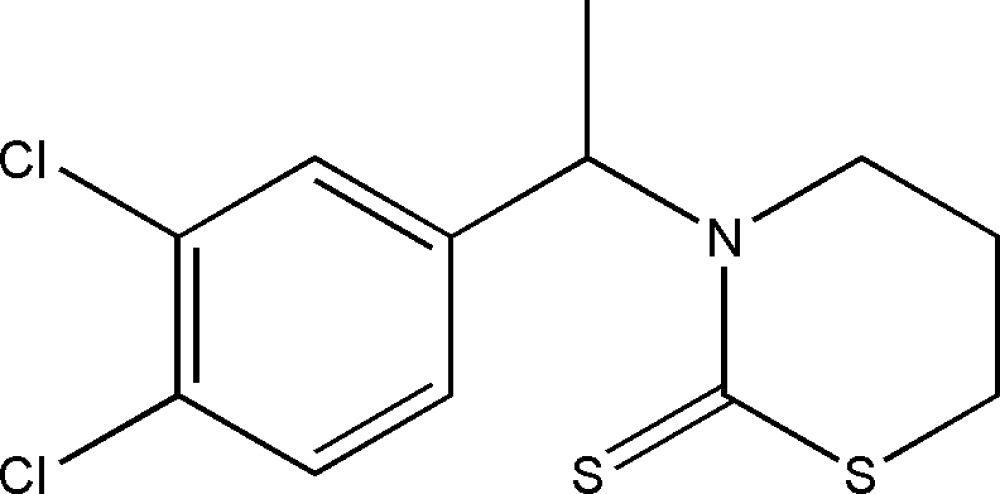


## Experimental

### 

#### Crystal data

C_12_H_13_Cl_2_NS_2_
*M* *_r_* = 306.25
Monoclinic, 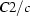

*a* = 13.6003 (13) Å
*b* = 6.7270 (7) Å
*c* = 29.149 (3) Å
β = 101.417 (4)°
*V* = 2614.1 (5) Å^3^
*Z* = 8
Mo *K*α radiation
μ = 0.79 mm^−1^
*T* = 113 K
0.14 × 0.12 × 0.08 mm

#### Data collection

Rigaku Saturn CCD area-detector diffractometer
Absorption correction: multi-scan (*CrystalClear*; Rigaku, 2005 ▶) *T*_min_ = 0.897, *T*_max_ = 0.939
11612 measured reflections
3029 independent reflections
2527 reflections with *I* > 2σ(*I*)
*R*_int_ = 0.048

#### Refinement

*R*[*F* ^2^ > 2σ(*F* ^2^)] = 0.035
*wR*(*F* ^2^) = 0.085
*S* = 1.07
3029 reflections
155 parameters
H-atom parameters constrained
Δρ_max_ = 0.32 e Å^−3^
Δρ_min_ = −0.27 e Å^−3^

### 

Data collection: *CrystalClear* (Rigaku, 2005 ▶); cell refinement: *CrystalClear*; data reduction: *CrystalClear*; program(s) used to solve structure: *SHELXTL* (Sheldrick, 2008 ▶); program(s) used to refine structure: *SHELXTL*; molecular graphics: *SHELXTL*; software used to prepare material for publication: *SHELXTL*.

## Supplementary Material

Crystal structure: contains datablocks I, global. DOI: 10.1107/S1600536809046352/rz2386sup1.cif

Structure factors: contains datablocks I. DOI: 10.1107/S1600536809046352/rz2386Isup2.hkl

Additional supplementary materials:  crystallographic information; 3D view; checkCIF report

## Figures and Tables

**Table 1:** Hydrogen-bond geometry (Å, °)

*D*—H⋯*A*	*D*—H	H⋯*A*	*D*⋯*A*	*D*—H⋯*A*
C5—H5⋯S1	1.00	2.48	3.068 (2)	117

**Fig. 1.**
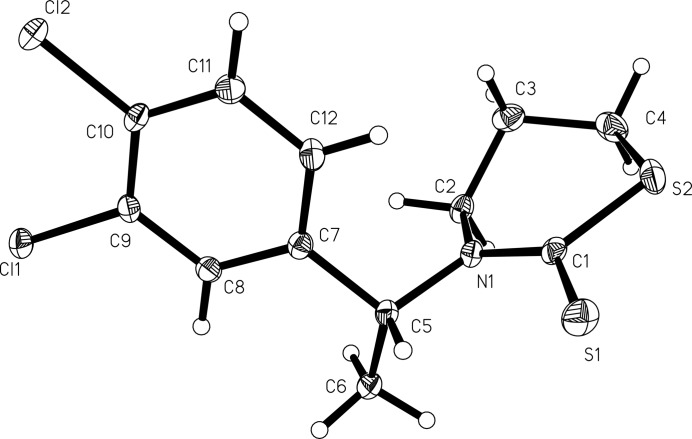
The molecular structure of the title compound, with displacement ellipsoids drawn at the 40% probability level.

## supplementary crystallographic information

### Comment

The 1,3-thiazinane ring is an important group in organic chemistry, as many compounds containing this groups possess a broad spectrum of biological activities (Soloway *et al.*, 1978; Tomizawa *et al.*, 1995). Herein, we report the crystal structure of the title compound, 3-[1-(3,4-dichlorophenyl)ethyl]-1,3-thiazinane-2-thione.

In title compound (Fig. 1), all bond lengths and angles are normal and in good agreement with those reported previously for the related compounds 2-phenylimino-1,3-thiazine (Kálmán, *et al.*, 1977) and (Z)-(1,3-thiazinan-2-ylideneamino)formonitrile (Peng & Wu, 2009). The thiazinane ring adopts a half boat conformation, with atom C3 displaced by 0.685 (2) Å from the plane (p1) formed by S2, N1, C1, C2 and C4 [maximum least squares plane deviation 0.040 (3) Å for N1]. The ring puckering parameters of the thiazinane ring are q2 = 0.512 (2) Å, θ = 130.1 (3)° and φ = 57.12 (2)° (Cremer & Pople, 1975). The dihedral angle between the benzene ring (C7—C12) and plane p1 is 84.18 (3)°. The molecular conformation is stabilized by an intramolecular C—H···S hydrogen bond (Table 1). In the crystal structure, centrosymmetrically related molecules at (*x*, *y*, *z*) and (-*x*, -*y*, -*z*) are linked by an aromatic π–π stacking interaction involving the benzene rings, with a centroid-to-centroid separation of 3.790 (2) Å.

### Experimental

A solution of 1,3-thiazinane-2-thione (1.33 g, 10 mmol) and sodium hydride (0.3 g) in anhydrous acetonitrile (20 ml) was added dropwise over a period of 10 min to a solution of 1,2-dichloro-4-(1-chloroethyl)benzene (2.10 g, 10 mmol) in acetonitrile (10 ml) at 273 K. The mixture was stirred at 353 K for 3 h. The solvent was removed and the residue was purified by flash chromatography (eluted with 5:1 v/v cyclohexane/dichloromethane) to give title compound as a white solid (2.66 g, 87% yield). Single crystals suitable for X-ray measurements were obtained by slow evaporation of an ethanol solution at room temperature.

### Refinement

All H atoms were placed in calculated positions, with C—H = 0.95–1.00 Å, and included in the final cycles of refinement using a riding model, with *U*_iso_(H) = 1.2*U*_eq_(C) or 1.5*U*_eq_(C) for methyl H atoms.

### Crystal data

**Table:**

C_12_H_13_Cl_2_NS_2_	*F*(000) = 1264
*M**_r_* = 306.25	*D*_x_ = 1.556 Mg m^−^^3^
Monoclinic, *C*2/*c*	Mo *K*α radiation, λ = 0.71073 Å
Hall symbol: -C 2yc	Cell parameters from 7182 reflections
*a* = 13.6003 (13) Å	θ = 2.3–27.5°
*b* = 6.7270 (7) Å	µ = 0.79 mm^−^^1^
*c* = 29.149 (3) Å	*T* = 113 K
β = 101.417 (4)°	Platelet, colourless
*V* = 2614.1 (5) Å^3^	0.14 × 0.12 × 0.08 mm
*Z* = 8	

### Data collection

**Table:**

Rigaku Saturn CCD area-detector diffractometer	3029 independent reflections
Radiation source: rotating anode	2527 reflections with *I* > 2σ(*I*)
confocal	*R*_int_ = 0.048
ω scans	θ_max_ = 27.8°, θ_min_ = 2.9°
Absorption correction: multi-scan (*CrystalClear*; Rigaku, 2005)	*h* = −17→17
*T*_min_ = 0.897, *T*_max_ = 0.939	*k* = −8→8
11612 measured reflections	*l* = −37→36

### Refinement

**Table:**

Refinement on *F*^2^	Primary atom site location: structure-invariant direct methods
Least-squares matrix: full	Secondary atom site location: difference Fourier map
*R*[*F*^2^ > 2σ(*F*^2^)] = 0.035	Hydrogen site location: inferred from neighbouring sites
*wR*(*F*^2^) = 0.085	H-atom parameters constrained
*S* = 1.07	*w* = 1/[σ^2^(*F*_o_^2^) + (0.0402*P*)^2^ + 0.4571*P*] where *P* = (*F*_o_^2^ + 2*F*_c_^2^)/3
3029 reflections	(Δ/σ)_max_ = 0.001
155 parameters	Δρ_max_ = 0.32 e Å^−^^3^
0 restraints	Δρ_min_ = −0.27 e Å^−^^3^

### Special details

**Table:**

Geometry. All e.s.d.'s (except the e.s.d. in the dihedral angle between two l.s. planes) are estimated using the full covariance matrix. The cell e.s.d.'s are taken into account individually in the estimation of e.s.d.'s in distances, angles and torsion angles; correlations between e.s.d.'s in cell parameters are only used when they are defined by crystal symmetry. An approximate (isotropic) treatment of cell e.s.d.'s is used for estimating e.s.d.'s involving l.s. planes.
Refinement. Refinement of *F*^2^ against ALL reflections. The weighted *R*-factor *wR* and goodness of fit *S* are based on *F*^2^, conventional *R*-factors *R* are based on *F*, with *F* set to zero for negative *F*^2^. The threshold expression of *F*^2^ > σ(*F*^2^) is used only for calculating *R*-factors(gt) *etc*. and is not relevant to the choice of reflections for refinement. *R*-factors based on *F*^2^ are statistically about twice as large as those based on *F*, and *R*- factors based on ALL data will be even larger.

### Fractional atomic coordinates and isotropic or equivalent isotropic displacement parameters (Å^2^)

**Table:**

	*x*	*y*	*z*	*U*_iso_*/*U*_eq_	
Cl1	0.60316 (4)	0.10311 (6)	0.452969 (16)	0.02326 (13)	
Cl2	0.63526 (3)	0.55467 (7)	0.429381 (15)	0.02298 (13)	
S1	0.55056 (4)	0.60336 (7)	0.693068 (19)	0.02744 (14)	
S2	0.75483 (4)	0.70607 (7)	0.725845 (17)	0.02484 (13)	
N1	0.69926 (10)	0.4226 (2)	0.66046 (5)	0.0155 (3)	
C1	0.66962 (14)	0.5552 (3)	0.68892 (6)	0.0177 (4)	
C2	0.80510 (13)	0.3723 (3)	0.66064 (7)	0.0189 (4)	
H2A	0.8097	0.3130	0.6300	0.023*	
H2B	0.8277	0.2709	0.6851	0.023*	
C3	0.87453 (14)	0.5495 (3)	0.66958 (7)	0.0256 (4)	
H3A	0.9428	0.5094	0.6662	0.031*	
H3B	0.8507	0.6541	0.6461	0.031*	
C4	0.87848 (15)	0.6317 (3)	0.71811 (7)	0.0281 (5)	
H4A	0.9241	0.7479	0.7232	0.034*	
H4B	0.9056	0.5294	0.7416	0.034*	
C5	0.62405 (13)	0.3052 (2)	0.62764 (6)	0.0162 (4)	
H5	0.5563	0.3438	0.6331	0.019*	
C6	0.63753 (15)	0.0842 (3)	0.63832 (7)	0.0224 (4)	
H6A	0.7011	0.0391	0.6309	0.034*	
H6B	0.5820	0.0101	0.6193	0.034*	
H6C	0.6382	0.0613	0.6716	0.034*	
C7	0.62876 (13)	0.3644 (3)	0.57774 (6)	0.0168 (4)	
C8	0.61792 (13)	0.2249 (3)	0.54197 (6)	0.0172 (4)	
H8	0.6098	0.0885	0.5488	0.021*	
C9	0.61883 (13)	0.2827 (3)	0.49638 (6)	0.0163 (4)	
C10	0.63158 (12)	0.4809 (3)	0.48584 (6)	0.0167 (4)	
C11	0.64194 (14)	0.6213 (3)	0.52126 (7)	0.0208 (4)	
H11	0.6504	0.7575	0.5144	0.025*	
C12	0.63994 (14)	0.5635 (3)	0.56657 (6)	0.0197 (4)	
H12	0.6463	0.6612	0.5905	0.024*	

### Atomic displacement parameters (Å^2^)

**Table:**

	*U*^11^	*U*^22^	*U*^33^	*U*^12^	*U*^13^	*U*^23^
Cl1	0.0312 (3)	0.0223 (2)	0.0161 (2)	−0.00385 (19)	0.00449 (19)	−0.00435 (18)
Cl2	0.0274 (3)	0.0267 (2)	0.0152 (2)	0.00102 (19)	0.00507 (19)	0.00504 (18)
S1	0.0251 (3)	0.0273 (3)	0.0333 (3)	0.0055 (2)	0.0140 (2)	−0.0023 (2)
S2	0.0338 (3)	0.0205 (2)	0.0181 (3)	0.0007 (2)	−0.0003 (2)	−0.00394 (18)
N1	0.0150 (7)	0.0191 (7)	0.0123 (8)	0.0016 (6)	0.0023 (6)	−0.0010 (6)
C1	0.0246 (9)	0.0159 (8)	0.0127 (9)	0.0017 (7)	0.0038 (7)	0.0034 (7)
C2	0.0166 (9)	0.0248 (9)	0.0158 (10)	0.0034 (7)	0.0046 (7)	−0.0008 (7)
C3	0.0190 (9)	0.0317 (10)	0.0259 (11)	−0.0019 (8)	0.0040 (8)	0.0052 (9)
C4	0.0245 (10)	0.0281 (10)	0.0279 (12)	−0.0067 (8)	−0.0038 (8)	0.0002 (8)
C5	0.0171 (8)	0.0185 (9)	0.0126 (9)	−0.0007 (7)	0.0016 (7)	−0.0002 (7)
C6	0.0307 (10)	0.0215 (9)	0.0152 (10)	−0.0041 (8)	0.0046 (8)	0.0015 (7)
C7	0.0146 (8)	0.0201 (8)	0.0151 (9)	0.0014 (7)	0.0018 (7)	0.0004 (7)
C8	0.0172 (8)	0.0163 (8)	0.0176 (10)	−0.0008 (7)	0.0024 (7)	0.0000 (7)
C9	0.0144 (8)	0.0197 (9)	0.0145 (9)	−0.0006 (7)	0.0022 (7)	−0.0039 (7)
C10	0.0153 (8)	0.0224 (9)	0.0125 (9)	0.0021 (7)	0.0028 (7)	0.0029 (7)
C11	0.0252 (10)	0.0162 (9)	0.0201 (10)	0.0010 (7)	0.0027 (8)	0.0017 (7)
C12	0.0227 (9)	0.0192 (9)	0.0160 (10)	0.0016 (7)	0.0011 (7)	−0.0023 (7)

### Geometric parameters (Å, °)

**Table:**

Cl1—C9	1.7317 (18)	C5—C7	1.522 (2)
Cl2—C10	1.7292 (18)	C5—C6	1.522 (2)
S1—C1	1.6789 (19)	C5—H5	1.0000
S2—C1	1.7430 (19)	C6—H6A	0.9800
S2—C4	1.811 (2)	C6—H6B	0.9800
N1—C1	1.334 (2)	C6—H6C	0.9800
N1—C2	1.478 (2)	C7—C8	1.389 (2)
N1—C5	1.483 (2)	C7—C12	1.393 (2)
C2—C3	1.511 (3)	C8—C9	1.387 (2)
C2—H2A	0.9900	C8—H8	0.9500
C2—H2B	0.9900	C9—C10	1.387 (2)
C3—C4	1.510 (3)	C10—C11	1.386 (2)
C3—H3A	0.9900	C11—C12	1.382 (3)
C3—H3B	0.9900	C11—H11	0.9500
C4—H4A	0.9900	C12—H12	0.9500
C4—H4B	0.9900		
			
C1—S2—C4	106.42 (9)	N1—C5—H5	107.4
C1—N1—C2	124.42 (15)	C7—C5—H5	107.4
C1—N1—C5	120.18 (15)	C6—C5—H5	107.4
C2—N1—C5	115.30 (13)	C5—C6—H6A	109.5
N1—C1—S1	126.05 (14)	C5—C6—H6B	109.5
N1—C1—S2	121.84 (14)	H6A—C6—H6B	109.5
S1—C1—S2	112.08 (10)	C5—C6—H6C	109.5
N1—C2—C3	113.25 (15)	H6A—C6—H6C	109.5
N1—C2—H2A	108.9	H6B—C6—H6C	109.5
C3—C2—H2A	108.9	C8—C7—C12	118.30 (17)
N1—C2—H2B	108.9	C8—C7—C5	121.49 (15)
C3—C2—H2B	108.9	C12—C7—C5	120.16 (16)
H2A—C2—H2B	107.7	C9—C8—C7	120.70 (16)
C4—C3—C2	110.84 (16)	C9—C8—H8	119.7
C4—C3—H3A	109.5	C7—C8—H8	119.7
C2—C3—H3A	109.5	C8—C9—C10	120.44 (16)
C4—C3—H3B	109.5	C8—C9—Cl1	118.80 (14)
C2—C3—H3B	109.5	C10—C9—Cl1	120.75 (14)
H3A—C3—H3B	108.1	C11—C10—C9	119.28 (16)
C3—C4—S2	110.86 (13)	C11—C10—Cl2	119.71 (14)
C3—C4—H4A	109.5	C9—C10—Cl2	121.02 (14)
S2—C4—H4A	109.5	C12—C11—C10	120.12 (17)
C3—C4—H4B	109.5	C12—C11—H11	119.9
S2—C4—H4B	109.5	C10—C11—H11	119.9
H4A—C4—H4B	108.1	C11—C12—C7	121.16 (17)
N1—C5—C7	108.84 (14)	C11—C12—H12	119.4
N1—C5—C6	110.40 (14)	C7—C12—H12	119.4
C7—C5—C6	115.16 (15)		
			
C2—N1—C1—S1	−174.57 (13)	C6—C5—C7—C8	17.1 (2)
C5—N1—C1—S1	1.7 (2)	N1—C5—C7—C12	−41.1 (2)
C2—N1—C1—S2	7.3 (2)	C6—C5—C7—C12	−165.64 (16)
C5—N1—C1—S2	−176.51 (12)	C12—C7—C8—C9	0.4 (3)
C4—S2—C1—N1	−2.87 (17)	C5—C7—C8—C9	177.67 (15)
C4—S2—C1—S1	178.74 (10)	C7—C8—C9—C10	0.6 (3)
C1—N1—C2—C3	−37.4 (2)	C7—C8—C9—Cl1	−179.05 (13)
C5—N1—C2—C3	146.26 (15)	C8—C9—C10—C11	−1.0 (3)
N1—C2—C3—C4	64.7 (2)	Cl1—C9—C10—C11	178.73 (13)
C2—C3—C4—S2	−58.89 (18)	C8—C9—C10—Cl2	178.77 (13)
C1—S2—C4—C3	28.40 (16)	Cl1—C9—C10—Cl2	−1.6 (2)
C1—N1—C5—C7	113.96 (17)	C9—C10—C11—C12	0.3 (3)
C2—N1—C5—C7	−69.49 (18)	Cl2—C10—C11—C12	−179.45 (14)
C1—N1—C5—C6	−118.73 (18)	C10—C11—C12—C7	0.7 (3)
C2—N1—C5—C6	57.82 (19)	C8—C7—C12—C11	−1.1 (3)
N1—C5—C7—C8	141.65 (16)	C5—C7—C12—C11	−178.40 (16)

### Hydrogen-bond geometry (Å, °)

**Table:**

*D*—H···*A*	*D*—H	H···*A*	*D*···*A*	*D*—H···*A*
C5—H5···S1	1.00	2.48	3.068 (2)	117
